# Spatial–Temporal Variation in Anthocyanidins in Novel Purple Corn (*Zea mays* L., *cv* Jizi-01)

**DOI:** 10.3390/plants15172601

**Published:** 2026-08-26

**Authors:** Cuicui Liu, Dongyang Li, Xue Bai, Dongfei Tan, Yunping Zhao, Xiaoming Chen, Ruixiang Yan, Pan Zou

**Affiliations:** 1 Institute of Agro-Product Safety and Nutrition, Tianjin Academy of Agricultural Sciences, Tianjin 300192, China; kedayanjiusheng@126.com (C.L.); 15764756869@163.com (D.L.); rosemary.324@163.com (X.B.); tandongfei1991@163.com (D.T.); yixi21504@163.com (Y.Z.); 2 College of Food Science and Bioengineering, Tianjin Agricultural University, Tianjin 300384, China; 3 Institute of Storage and Process for Agro-Product, Tianjin Academy of Agricultural Sciences, Tianjin 300192, China; chxm001@126.com; 4 College of Light Industry Science and Engineering, Tianjin University of Science and Technology, State Key Laboratory of Biobased Fiber Materials, Tianjin 300457, China

**Keywords:** anthocyanidins, purple corn, spatial-temporal variation, antioxidant activity

## Abstract

Purple corn is an anthocyanidin-rich plant, with anthocyanidins being particularly abundant in the waste it produces. This study explored the variation in anthocyanidins in each part of the plant during the growth period of a novel type of purple corn (*Zea mays* L., *cv* Jizi-01). The results showed that vacuum freeze-drying had advantages over cabinet drying in protecting anthocyanidins, except in the cornsilk part. The anthocyanidin contents of the different plant parts varied with time. Purple corn cob anthocyanidins (PCCAs) were isolated and identified owing to their abundance (14.95 g/kg, dry basis (DB)) among all plant parts. PCCA comprises three anthocyanidins, namely cyanidin, pelargonidin and peonidin, forming 11 types of anthocyanins when combined with glycosidic bonds. Finally, the in vitro scavenging effects of PCCA on DPPH, ABTS·^+^ and ·OH radicals were evaluated, and the scavenging rates reached up to 89.92%, 96.95% and 90.76%. This study shows that purple corn (*Zm* Jizi-01) is a novel resource rich in anthocyanidins, with high economic potential.

## 1. Introduction

As a typical class of flavonoids, anthocyanidins are widely distributed in berries, grapes, purple yams, purple corn, black rice, and other dark plants [1,2]. Anthocyanidins are often used as food pigments to prepare drinks, jellies, and candies [3,4,5]. Moreover, anthocyanidins have attracted increasing attention as functional active ingredients because of their various health-promoting activities, including antioxidant and anti-tumor effects [6,7,8]. Natural anthocyanidins from fruits and vegetables are expensive due to the high cost of the raw materials. Therefore, it is viable to isolate anthocyanidins from biological wastes.

Purple corn is an abundant and economically viable source of anthocyanidins, especially in corn cobs, which are a by-product [9,10]. Simple anthocyanidins mainly exist in the aleurone layer of corn endosperm or pericarp and greatly affect the color of the seeds. In addition, the abundant anthocyanidins in husks, cobs, and silk are by-products of purple corn and show great potential for the extraction of natural anthocyanidins [11]. However, purple corn by-products are commonly used to make silage or firewood after harvest, making them a waste of anthocyanidin resources [12]. Therefore, in-depth research can provide a scientific basis for the comprehensive utilization of purple corn by-products. Previous studies have established that anthocyanidins (e.g., cyanidin, pelargonidin, and peonidin) combined with glycosidic bonds form various anthocyanins as the predominant structural derivatives of purple corn [13], and cyanidin-3-glucoside (C3G) is one of the most common anthocyanins in the majority of purple corn [14]. Pelargonidin-3-glucoside (Pr3G) and peonidin-3-glucoside (Pn3G) are widely found in cobs, bran, husks, and kernels of purple corn. Additionally, other glycoside derivatives were also identified in the husks of purple corn from Mexico and the cob of purple corn from China, including pelargonidin-3-(6″-malonylglucoside), cyanidin-3-(6″-malonylglucoside), and peonidin-(6″-malonylglucoside) [15]. Furthermore, the anthocyanidin levels of purple corn differ according to variety and origin. Combined with traditional breeding techniques, phenotypic selection and hybridization, a number of purple corn varieties with high anthocyanidins, such as ‘Morado’, ‘Apache Red Cob’, ‘Heizhenzhu’, ‘Zihei’, and ‘Supersweet’, have been identified [16]. It has been reported that there were 3.07 mg/g, 7.21 mg/g, and 7.54 mg/g dry weight of C3G in purple corn from the United States, Mexico, and Bolivia, respectively [17].

Previous studies have primarily focused on anthocyanidin content at the final harvest stage, whereas the dynamic accumulation patterns and profiles of anthocyanidins throughout the growth period remain largely unexplored. To make full use of the anthocyanidins in purple corn, it is necessary to clarify the anthocyanidin composition and changing rules during the growth period. In this study, a novel purple corn cultivar, *Zm* Jizi-01, was studied, which contains a considerable amount of anthocyanidins compared to previously published purple corn cultivars [16,18,19,20,21,22]. The anthocyanidin contents were analyzed at the different growth stages of various parts and were then isolated and purified from the corn cobs. Moreover, the composition of PCCA was identified using high-performance liquid chromatography (HPLC) and ultra-performance liquid chromatography–quadrupole time-of-flight mass spectrometry (UPLC-QTOF-MS), and Fourier transform infrared spectroscopy (FT-IR), and the antioxidant effects of PCCA were explored against several free radicals.

## 2. Results and Discussion

### 2.1. The Effects of Drying Methods on Anthocyanidins

**Figure 1:**
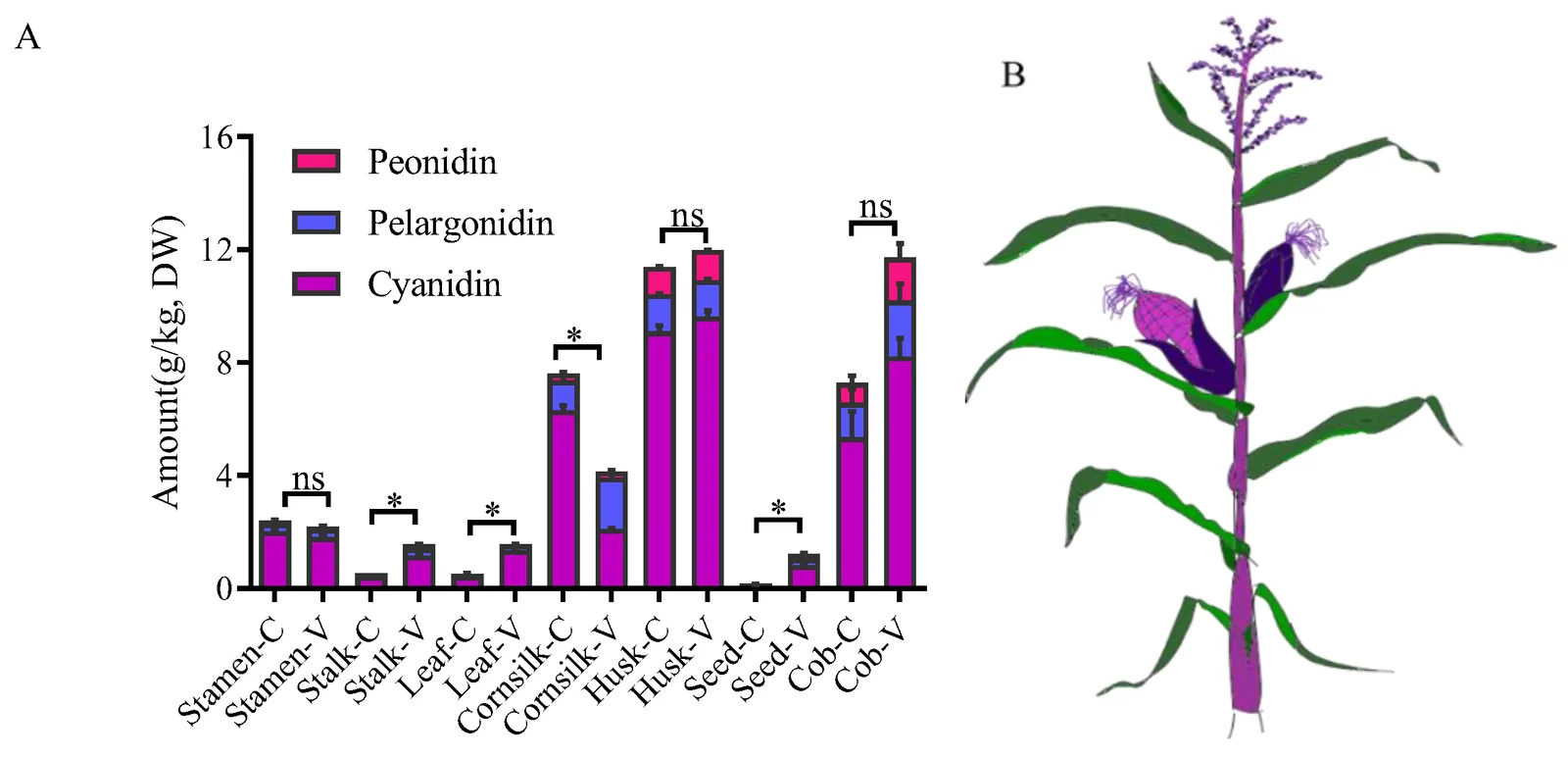
Anthocyanidin contents (g/kg dry basis, DB) in different parts of the novel purple corn *Zm* Jizi-01. (**A**) Anthocyanidin compositions and amounts in different parts (kernel, cob, cornsilk, husk, leaf and stalk) of purple corn collected at Day 110 after sowing (13 May 2020) and extracted with a mixture of ethanol, water, and hydrochloric acid (2:1:1, *v*/*v*/*v*) by two drying methods. C for cabinet drying, and V for vacuum freeze drying. (**B**) Distribution diagram of anthocyanidin content of purple corn; shade of purple represents the level of anthocyanidin content. Statistical comparisons among different drying treatment groups were performed using one-way analysis of variance (ANOVA); ‘*’ indicates a significance level of *p* < 0.05, and ‘ns’ means no significance.

Different parts of the purple corn were dried using cabinet drying or vacuum freeze drying. Anthocyanidins were isolated and analyzed using HPLC. There were three anthocyanidins in *Zm* Jizi-01, including cyanidin, pelargonidin, and peonidin, but peonidin was absent in the leaves, as shown in Figure 1A. The data showed that the vacuum freezing treatment protected anthocyanidins in most parts, except for cornsilk. The total anthocyanidin contents in cornsilk obtained by cabinet drying were significantly higher than those obtained by vacuum freeze drying (*p* < 0.05). The highest total amount following vacuum freeze drying was found in the husk (11.974 g/kg, DB), followed by the cob (11.711 g/kg, DB), cornsilk (6.382 g/kg, DB), stamen (2.198 g/kg, DB), leaf (1.573 g/kg, DB), stalk (1.565 g/kg, DB), and seed (1.229 g/kg, DB). Figure 1B shows the changes in the contents of different parts of the purple corn. The darker the color, the higher the anthocyanidin content. Vacuum-frozen seeds were peeled to obtain corn bran, which contained all the anthocyanidins (1.226 g/kg, DB).

It is well known that anthocyanidins are sensitive to pH, heat, oxygen, and other factors [23,24]. Therefore, cabinet drying may destroy the anthocyanidin structure. A similar observation was made in blueberries dried by programmed heating, in which the total anthocyanidin content was less than that of fresh berries [25]. Sharma et al. have also found that heating at 105 °C for varied times decreased the total anthocyanidin content in *Eugenia jambolana* (jamun) extract [26]. Continued heating resulted in the opening of the B ring of anthocyanidin and its further degradation to phloroglucinaldehyde or other phenolic acids [27]. Moreover, it was reported that mild heat treatment up to 50 °C may inactivate the enzymatic reaction in raw material and prevent phenolic compounds from being degraded by polyphenol oxidase [28], which offers a possible reason for the increased content in cornsilk.

### 2.2. Anthocyanidin Changes During the Growth Period

During the early growth stages of purple corn, the anthocyanidin content may be relatively low, as the metabolic activity of the plant is mainly focused on basic growth and development. Therefore, we collected different parts of the purple corn on Days 70, 80, 90, 110, and 120 after planting. Seven parts of the purple corn were collected, including the stamen, leaf, stalk, cornsilk, husk, seed, and cob. As shown in Table 1, the total anthocyanidin contents in the stamen, leaf, stalk, and cornsilk increased over time from Day 70 to Day 110. The total anthocyanidin contents in the husk and cob also accumulated. Spatially, the anthocyanidin content was the highest in husks from Day 80 to Day 110 (Table 1). On Day 120, the anthocyanidin content in the husk decreased, indicating that as the harvest time approached, the purple corn plants began to lose water and wither. On Days 70 and 80, anthocyanidins were not detected in the leaves, but were detected in the stalk and husk. Anthocyanidins mainly accumulate in stalks and husks at maturity, reaching their highest level at 110 and 120 days after sowing, respectively. This observation indicates a temporal correlation between developmental stage and anthocyanidin accumulation under the field conditions tested. In addition, anthocyanidin synthesis is affected by various environmental factors, such as light, temperature, and soil conditions, as well as the maturity of each part of the plant [29]. While these conditions affect the overall metabolism of plants, they may also affect the accumulation of anthocyanidins in different plant parts, and the cob is more conducive to the accumulation of anthocyanidins because of its structure and location. Spatial and temporal variation analyses of anthocyanidins in *Zm* Jizi-01 revealed the optimal harvest window for accumulation and established a comprehensive balance between the competing demands of industrial extraction and silage utilization, which offers a useful reference for agricultural and by-product management.

### 2.3. Optimized Isolation of Anthocyanidins from Cobs

**Figure 2:**
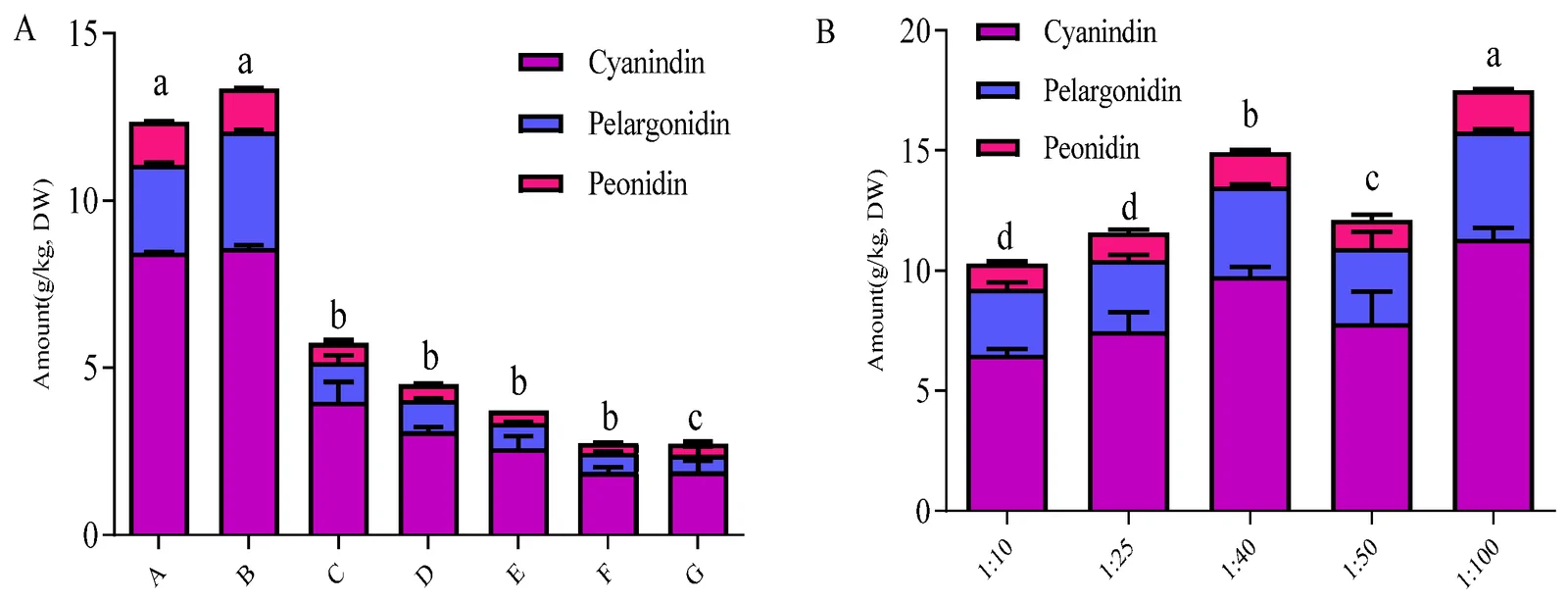
Effects of different extraction conditions on the extraction of anthocyanidin. (**A**) Different extract solutions: extract solution A: ethanol: water: hydrochloric acid = 2:1:1 (*v*/*v*/*v*); extract solution B: 95% ethanol: water: hydrochloric acid = 2.10:1:0.9 (*v*/*v*/*v*); extract solution C: ethanol: water: hydrochloric acid = 2:2:1 (*v*/*v*/*v*); extract solution D: ethanol: water: hydrochloric acid = 3:3:1 (*v*/*v*/*v*); extract solution E: ethanol: water: hydrochloric acid = 4:2:1 (*v*/*v*/*v*); extract solution F: ethanol: water: hydrochloric acid = 4:4:1 (*v*/*v*/*v*); extract solution G: ethanol: water: hydrochloric acid = 2:1:0.3 (*v*/*v*/*v*). (**B**) Different solid–liquid ratios. Solid–liquid ratio of cob powder and extract solution B at 1:10, 1:25, 1:40, 1:50 and 1:100. Statistical comparisons among different groups were performed using one-way analysis of variance (ANOVA); different lowercase letters on each bar indicate significant differences (*p* < 0.05) among different extractions.

Since the proportion of husk in purple corn is relatively smaller than the cob, which contains considerable anthocyanidins (in Table 1), we optimized the isolation process of anthocyanidins from the cob for further study. In this study, the extraction solution and solid–liquid ratio were optimized. As shown in Figure 2A, the amount of anthocyanidins obtained by the seven groups of extracts were 12.36 ± 0.13 g/kg DB (A), 13.36 ± 0.17 g/kg DB (B), 5.76 ± 1.20 g/kg DB (C), 4.51 ± 0.27 g/kg DB (D), 3.62 ± 0.37 g/kg DB (E), 2.76 ± 0.23 g/kg DB (F), and 2.74 ± 0.28 g/kg DB (G), respectively. There was no difference between the anthocyanidin contents of groups A and B (*p* > 0.05), while the anthocyanidin contents of the other extracts were significantly lower than that of group A (*p* < 0.01). Moreover, there were some impurity peaks in the chromatograms, owing to insufficient acid hydrolysis. The acid used in the anthocyanidin extraction is important in preventing anthocyanidin degradation. The hydrochloric acid contents of the seven groups were 9.25% (A), 9.25% (B), 7.40% (C), 6.16% (D), 5.29% (E), 4.11% (F), and 3.36% (G), respectively. As a high-fiber material, the anthocyanidin extract from purple corn cob required concentrated hydrochloric acid, and the ethanol and hydrochloric acid contents of the extract in group B were the same as those of group A. However, the cost of 95% ethanol is lower than that of absolute ethanol; therefore, the extract solution of group B was selected for PCCA extraction, that is, 95% ethanol: water: concentrated hydrochloric acid = 2.1:0.9:1 (*v*/*v*/*v*).

Next, we compared the effect of the solid–liquid ratio on anthocyanidin extraction. As shown in Figure 2B, the anthocyanidin yield obtained with a solid–liquid ratio of 1:100 was the highest at 17.51 ± 0.60 g/kg, which was 1.17 times that obtained by a solid–liquid ratio of 1:40 (14.95 ± 0.55 g/kg). However, the extraction solution volume used was 2.50 times greater, greatly increasing the extraction cost. In summary, a solid–liquid ratio of 1:40 was selected for subsequent extraction. PCCA was defined as the anthocyanidins extracted from purple corn (*Zm* Jizi-01) cobs using extract solution B at a solid–liquid ratio of 1:40.

### 2.4. Composition of Anthocyanidins in Purple Corn Cobs

HPLC was used to accurately separate and quantitatively analyze the anthocyanidin content of each part of purple corn, and the anthocyanidin contents in the husk, cob, and cornsilk of purple corn were significantly higher than those in the other parts (see Table 1). Among these, corn cobs account for a relatively large weight proportion in corn waste and are easier to obtain and collect. Moreover, PCCA offers new ideas for the comprehensive utilization of purple corn waste, provides the possibility for the development of cheap anthocyanidins, and possesses important economic significance and social value. Therefore, in a follow-up study, our group focused on anthocyanidins extracted from cobs. The anthocyanidin composition of corn cobs was analyzed after harvesting and drying. There were three anthocyanidins in the cob of purple corn, which were cyanidin (9.77 ± 0.39 g/kg DB), pelargonidin (3.74 ± 0.07 g/kg DB), and peonidin (1.43 ± 0.08 g/kg DB). The total anthocyanidin content was 14.95 g/kg DB. Although purple corn has been extensively investigated, anthocyanidin content varies tremendously among different genotypes. The novel variety of purple corn (*Zm* Jizi-01) used in this study exhibited a remarkably high total anthocyanidin content in its cob, which is higher than that reported in the literature in recent years and presents an ideal raw material for extracting anthocyanidins [30,31,32]. These findings indicate a highly promising new source for natural anthocyanins.

UPLC-QTOF-MS has shown great superiority in the quantitative analysis of anthocyanidins in complex samples, and the application of this technology is of great significance for the extraction, separation, and qualitative and quantitative analysis of anthocyanidins. In this study, UPLC-QTOF-MS was used to rapidly and accurately identify anthocyanins in a novel corn cob of purple corn. Eleven anthocyanin compounds were tentatively identified, specifically C3G, peonidin-3-glucoside (Pn3G), pelargonidin-3-glucoside (Pr3G), cyanidin-3-(dimalonylglucoside), cyanidin-3-(6′-malonylglucoside), cyanidin-5-(6′-malonylglucoside), cyanidin-7-(6′-malonylglucoside), peonidin-3-(dimalonylglucoside), peonidin-3-(6′-malonylglucoside), pelargonidin-3-(dimalonylglucoside), and pelargonidin-3-(6′-malonylglucoside). Among them, seven anthocyanins, C3G, Pr3G, Pn3G, cyanidin-3-(6″-malonylglucoside), pelargonidin-3-(6″-malonylglucoside), peonidin-3-(dimalonylglucoside), and peonidin-3-(6″-malonylglucoside), were identified in purple corn [6,33,34,35,36]. Surprisingly, four putative anthocyanins were ascribed in purple corn for the first time, including cyanidin-3-(dimalonylglucoside), cyanidin-5-(6″-malonylglucoside), cyanidin-7-(6″-malonylglucoside), and pelargonidin-3-(dimalonylglucoside). These results enrich our understanding of the chemical diversity of purple corn anthocyanins and offer new chemical entities for future biological activity research.

UPLC-QTOF-MS analysis characterized the native anthocyanin glycoside profile, whereas HPLC quantified the total hydrolyzable anthocyanin content expressed as aglycones. UPLC-QTOF-MS analysis revealed that the native anthocyanins in the purple corn cob were predominantly glycosides of cyanidin, pelargonidin, and peonidin (Table 2). Following acid hydrolysis, HPLC quantification confirmed that these three aglycones accounted for most of the hydrolyzable anthocyanin content. Thus, the UPLC-MS identification of the glycoside precursors is in good agreement with the HPLC quantification of their corresponding aglycones.

### 2.5. Structural Elucidation of Anthocyanidins in Purple Corn Cobs

**Figure 3:**
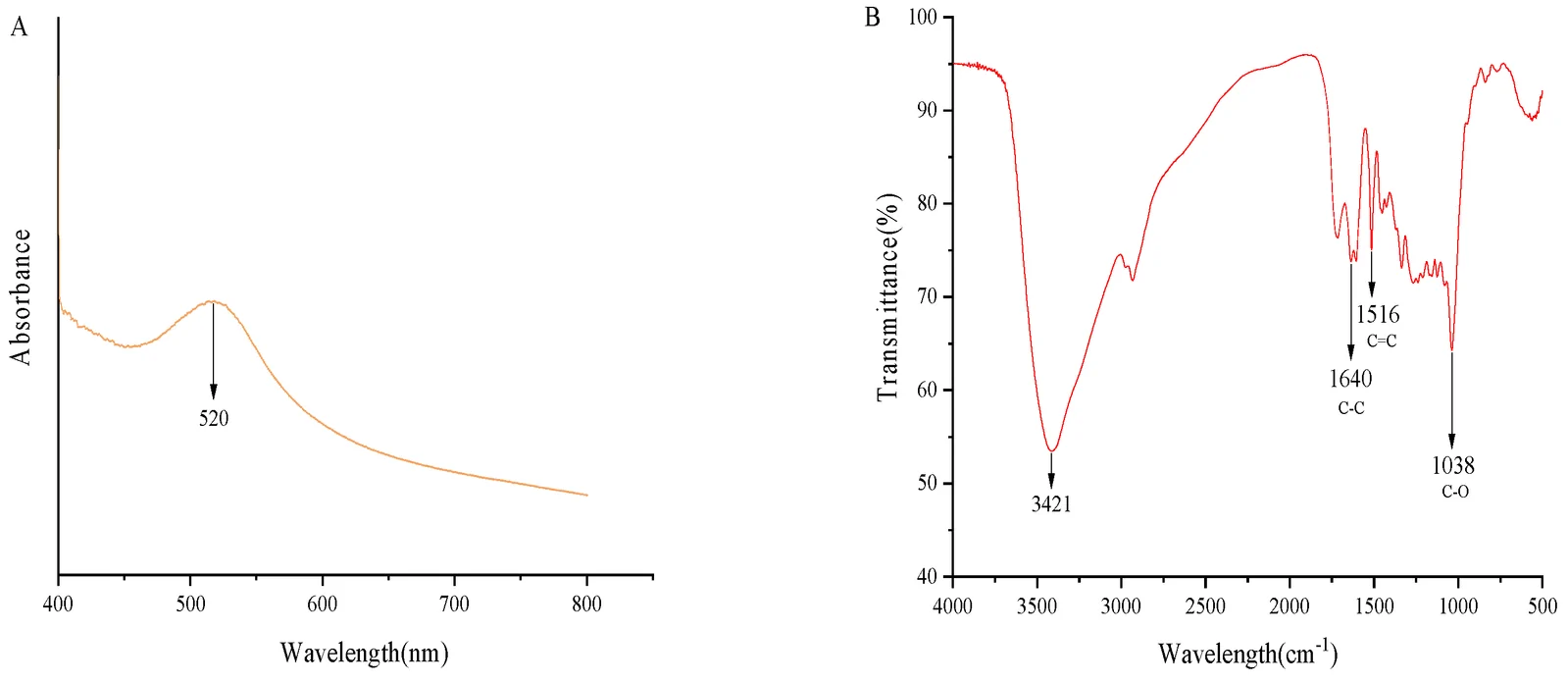
Spectra figures of PCCA. (**A**) UV-vis spectra. (**B**) FT-IR spectra.

In this study, we performed a detailed analysis of PCCA using UV-vis (Figure 3A). The results showed that PCCA exhibited significant absorption characteristics in the wavelength range of 400–800 nm. Specifically, the maximum absorption peak was observed at 520 nm, which is the characteristic absorption wavelength of anthocyanidin. This result is consistent with those of previous studies [37].

Anthocyanidins in purple corn exhibited unique absorption characteristics in their FT-IR spectra [38]. Specifically, a distinct absorption peak at 3421 cm^−1^ was attributed to the tensile vibration of the hydroxyl group (O–H). At 1640 cm^−1^ and 1038 cm^−1^, two absorption peaks were related to the tensile vibration of C–C and C–O in the aromatic ring framework, respectively (Figure 3B). The absorption peak at 1516 cm^−1^ was caused by the stretching of the aromatic ring of benzopyran, which further confirms the typical structural characteristics of PCCA as a flavonoid compound [39].

### 2.6. Antioxidant Capacity Assay of PCCA In Vitro

**Figure 4:**
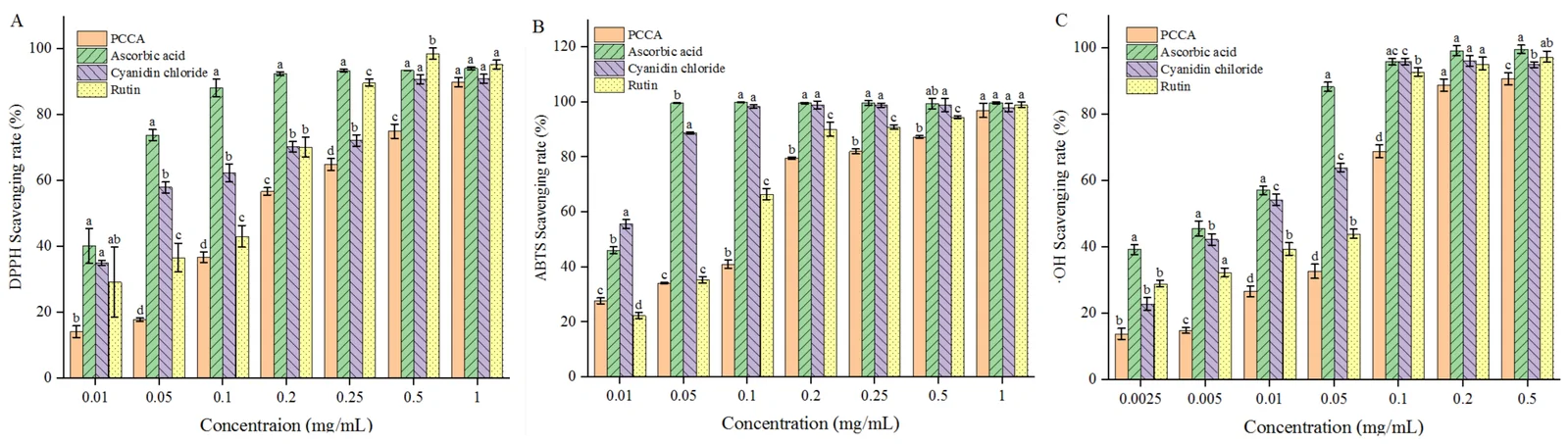
Scavenging effects of PCCA on DPPH (**A**), ABTS·^+^ (**B**), and ·OH (**C**) free radicals. PCCA is for the anthocyanidins extracted from corn cob, and ascorbic acid, cyanidin, and rutin were applied as positive controls for antioxidant effects. Different letters indicate statistical differences between different treatments.

This study performed DPPH, ABTS·^+^ and ·OH radical scavenging experiments to comprehensively evaluate the antioxidant capacity of PCCA in vitro [40]. As shown in Figure 4, ascorbic acid and cyanidin chloride (purity of 97.1%) generally showed higher scavenging activities at low concentrations, followed by rutin and PCCA. Compared to these substances, PCCA had a significant effect on DPPH, ABTS·^+^ and ·OH radicals. The scavenging rates of the three free radicals were significantly improved and were as high as 89.92 ± 1.41%, 96.95 ± 2.62% and 90.76 ± 1.75%, respectively, at higher concentrations (Figure 4). As the concentration of the sample solution increased, the scavenging rate of the three free radicals increased for each sample. When the sample concentration reached a certain value, the scavenging effect of each sample on the free radicals exhibited a certain saturation trend (Figure 4).

Additionally, we calculated the IC_50_ values of PCCA, ascorbic acid, cyanidin chloride, and rutin against the three free radicals to evaluate the scavenging effects of these natural products. The IC_50_ values of PCCA against DPPH, ABTS·^+^ and ·OH radicals were 0.159 mg/mL, 0.079 mg/mL, and 0.048 mg/mL, respectively (Table 3), which were slightly lower than those of rutin as a natural antioxidant (*p* < 0.05). These results clearly showed that PCCA had an in vitro antioxidant capacity close to that of rutin. The positive control group had the lowest IC_50_ value of ascorbic acid in the DPPH and ·OH free radical scavenging assays, indicating that it had the strongest antioxidant activity against the two radicals, while cyanidin chloride had the lowest IC_50_ value of 0.008 mg/mL in the ABTS·^+^ free radical scavenging assay, showing extremely strong antioxidant activity (Table 3). In summary, these data not only reflect their significant effect on antioxidant activity but also further highlight the importance of PCCA as a natural antioxidant, lay a foundation for further research on the antioxidant mechanism and application prospects of these compounds, and help to develop new antioxidative products.

## 3. Materials and Methods

### 3.1. Materials and Reagents

The purple corn (*Zea mays* L.) (*Zm* Jizi-01) used in this study was provided by the research group of the Maize Breeding Laboratory, Institute of Crop Sciences, Tianjin Academy of Agricultural Sciences, China. After sowing on 13 May 2020, the corn plants of *Zm* Jizi-01, grown in Jizhou District, Tianjin, China, were collected on Days 70, 80, 90, 110, and 120. Five plants were collected at each time and pooled into a single composite sample of each tissue type. All fresh parts, including the stamen, cornsilk, leaf, stalk, husk, seed, and cob, were divided into two parts; one part was placed in a hot-air drying oven (DHG-9140A, Shanghai Yiheng Instrument Co., Ltd., Shanghai, China), and the other part was first pre-frozen and then subjected to sublimation under reduced pressure (SCIENTZ-10N/D, Ningbo Scientiz Biotechnology Co., Ltd., Ningbo, China) at −80 °C under 5 Pa. Materials were ground until a constant weight was achieved and then sifted through 60 mesh sieves.

Delphinidin chloride (91.3%, HPLC), petunidin chloride (99.9%, HPLC), and cyanidin chloride (98.6%, HPLC) standards used for anthocyanidin composition analysis were purchased from First Standard, Alta Scientific Co. Ltd., Tianjin, China. Malvidin chloride (97.0%, HPLC), pelargonidin chloride (96.0%, HPLC), and peonidin chloride (97.0%, HPLC) used for anthocyanidin analysis were purchased from ANPEL Laboratory Technologies Inc., Shanghai, China. The positive controls in the scavenging assays, such as cyanidin chloride (97.1%, HPLC), rutin (95%, HPLC), DPPH (97%, HPLC), and ABTS·^+^ reagents (98%, HPLC), were purchased from ANPEL Laboratory Technologies Inc., Shanghai, China. Ascorbic acid (99.8%, HPLC) was obtained from Sinopharm Chemical Reagent Co., Ltd., Beijing, China. Acetonitrile was purchased from Beijing Dima Technology Co., Ltd., Beijing, China. All other reagents used were of the highest quality available.

### 3.2. Extraction of Anthocyanidins from Purple Corn

Anthocyanidins were extracted following the method described by Chen [41], with minor modifications. The sample powder (1.0 g) was suspended in a 50 mL mixture of ethanol, water, and hydrochloric acid (2:1:1, *v*/*v*/*v*). The samples were subjected to ultrasonic treatment at 300 W of ultrasonic power in the dark for 30 min at room temperature, and then hydrolyzed in a boiling water bath for one hour. After cooling down, the supernatant was filtered and subjected to HPLC analysis. The results were calculated as aglycones against commercial standards. All anthocyanidin contents are expressed on a dry basis (DB).

### 3.3. Isolation and Purification of Anthocyanidins from Purple Corn Cobs

Based on raw material and total anthocyanidin content, we optimized the extract solution and solid–liquid ratio to isolate anthocyanidins from corn cobs [42,43,44]. The mixtures of ethanol, water, and hydrochloric acid were 2:1:1(A), 2:2:1(C), 3:2:1(D), 4:2:1(E), 4:4:1(F), and 2:1:0.3(G), respectively. Then, a mixture (solution B) of 95% ethanol, water, and hydrochloric acid was also prepared to have the same concentrations of ethanol and hydrochloric acid as solution A. Cob powder was separately extracted by the solutions above and then treated as described in Section 3.2.

To optimize the solid–liquid ratio, 1 g of cob powder was suspended in different volumes of the selected extract solution, making solid–liquid ratios of 1:10, 1:25, 1:40, 1:50 and 1:100. Other treatments were the same as those described in Section 3.2.

Next, 100 g of cob powder was treated using the optimized extraction process. The supernatant obtained was filtered and concentrated at 50 °C via rotary evaporation. The crude extract was loaded onto a pre-equilibrated microporous resin D101 column (5.0 × 100.0 cm), and then sequentially eluted with 2 L of 95% ethanol and 2 L of distilled water. The eluent was collected according to its color. The magenta liquid was concentrated and lyophilized to obtain purple corn cob anthocyanidins, referred to as PCCA.

### 3.4. Determination of Anthocyanidin Composition and Content Using HPLC

Six anthocyanidin standards were dissolved in 10% hydrochloric acid–methanol. The six anthocyanidins and their mixtures were separately loaded to an HPLC system (e2695, Waters Co., Milford, MA, USA) equipped with a 2998 PDA detector. The samples were gradient-eluted with 1% formic acid in water and 1% formic acid in acetonitrile. The flow rate was 0.8 mL/min at 30 °C. The anthocyanidin composition and content were measured based on the retention time and peak area of the standards.

### 3.5. Identification of Anthocyanin Using UPLC-QTOF-MS

First, 5 mg of PCCA was dissolved in hydrochloric acid–methanol, and 15 μg of peonidin-3-glucoside chloride (Pn3G) was added as an internal standard. The mixture was then filtered for further analysis. UPLC-QTOF-MS was applied to the nonhydrolyzed native extract to profile the intact anthocyanin glycosides, with a few modifications [45]. A Waters ACQUITY UPLC I-Class-Xevo G2-XS QTOF/PDA lambda detector was used in combination with a CSH C18 column (2.1 mm × 100 mm, 1.7 μm). The samples were gradient eluted with 1% formic acid in acetonitrile and 1% formic acid in water at 0.15 mL/min. The mass spectrometer was set in positive ion mode for anthocyanin identification. The electrospray ionization source parameters were set as follows: electrospray capillary voltage, 2.0 kV; source temperature, 100 °C; desolvation temperature, 300 °C; desolvation gas flow, 800 L/h; cone gas flow, 50 L/h. Mass acquisition was performed using Fast DDA for 30 min. The analyzer was operated in resolution mode. The precursor molecular ion mass was acquired over the range from 900 *m*/*z* to 1000 *m*/*z* in continuum mode. The MS/MS for fragment spectra was over the range from 50 *m*/*z* to 1000 *m*/*z* in continuum mode. A maximum of five MS/MS ions were acquired for identification. A collision ramp was used to obtain the fragments. The ramp range was from 6 V to 80 V. Compound identification was conducted by matching against the Waters UNIFI Natural Products library, followed by manual verification of the precursor ions and characteristic fragment ions.

### 3.6. Spectrum Analysis

An ultraviolet-visible (UV-vis) spectrophotometer (PerkinElmer Lambda 365, PerkinElmer Inc., Co. Ltd., Waltham, MA, USA) was used. Briefly, PCCA powder was dissolved in distilled water and stirred until well dissolved. Then, the samples were placed in a 5 cm × 1 cm × 1 cm glass cuvette and scanned at 400–800 nm.

The chemical structure of PCCA was determined using Fourier-transform infrared spectroscopy (Nicolet 6700 FT-IR spectrometer, Nicolet., Co. Ltd., Madison, WI, USA). Then, PCCA was mixed with potassium bromide at a ratio of 1:100 (*w*/*w*) and pressed into pellets. The wavenumber range was 400–4000 cm^−1^, the spectrometer resolution was 4 cm^−1^, the signal-to-noise ratio was 50,000:1, and the scans were performed 32 times.

### 3.7. Antioxidant Activities of Anthocyanidins from Corn Cobs

#### 3.7.1. DPPH Scavenging Activity

We prepared sample solutions of 0.01 mg/mL, 0.05 mg/mL, 0.1 mg/mL, 0.2 mg/mL, 0.25 mg/mL, 0.5 mg/mL, and 1 mg/mL, referring to the method of Zou [46], with minor modifications. Then, 50 μL of the sample solution to be measured was mixed with 150 μL of 0.2 mM DPPH solution, and the absorbance was measured using a microplate reader at a wavelength of 517 nm with ascorbic acid, rutin, and cyanidin chloride as the positive controls. Equation (1) was used to calculate the scavenging rate.

Scavenging Rate (%) = [1 − (*A*_1_ − *A*_2_)/*A*_0_] × 100% (1)

where *A*_0_ is the absorbance of the control group, *A*_1_ represents the sample absorbance, and *A*_2_ represents the absorbance of the standard solution without an added sample.

#### 3.7.2. ABTS·^+^ Scavenging Activity

We prepared sample solutions of 0.01 mg/mL, 0.05 mg/mL, 0.1 mg/mL, 0.2 mg/mL, 0.25 mg/mL, 0.5 mg/mL, and 1 mg/mL, referring to Zhao [47], with modification. Then, 20 μL of different concentrations of the sample solution to be measured were mixed with 200 μL of ABTS·^+^ solution, and the absorbance was measured using a microplate reader at 734 nm at room temperature in the dark. Equation (1) was used to calculate the scavenging rate.

#### 3.7.3. ·OH Scavenging Activity

Sample solutions of 2.5 mg/L, 5 mg/L, 10 mg/L, 50 mg/L, 100 mg/L, 200 mg/L, and 500 mg/L were prepared as described by Jan [48], with modifications. Then, 0.1 mL of FeSO_4_ solution, 0.1 mL of salicylic acid–ethanol solution, and 1 mL of sample extraction solution were added to the tube. Finally, 0.1 mL of hydrogen peroxide solution was added to start the reaction, and the absorbance was measured by a microplate reader at a wavelength of 510 nm with ascorbic acid, rutin, and cyanidin chloride in a water bath at 37 °C for 1 h. Equation (1) was used to calculate the scavenging rate.

### 3.8. Statistical Analysis

All experimental data were expressed as mean ± standard deviation based on three independent replicates. The IC_50_ values of all treatments were calculated by GraphPad Prism 8.0. Statistical comparisons among groups were performed using one-way analysis of variance (ANOVA) at a significance level of *p* < 0.05, followed by Turkey’s range test for multiple comparisons.

## 4. Conclusions

In this study, the spatial–temporal variation, composition, structure, and antioxidant activity of anthocyanidins in purple corn (*Zm* Jizi-01) are discussed. We successfully isolated and purified anthocyanidins from purple corn cob and characterized their structure using UV-vis and FT-IR spectroscopy. The results showed that the anthocyanidin contents of different parts of purple corn changed with time and space during the growth cycle. The anthocyanidin content in the husk was the highest, but the anthocyanidin content gradually decreased as the harvest period approached. In addition, we found that purple corn cob is an ideal material for anthocyanidin extraction, because it contains a considerable content of anthocyanidins. Using the UPLC-QTOF-MS technique, we successfully identified 11 anthocyanins, among which 7 have been found in other purple corn varieties, and 4 were discovered in purple corn for the first time. Additionally, we evaluated the antioxidant activity of PCCA, and the results showed that PCCA had a positive scavenging effect on free radicals and an antioxidant capacity like that of the natural antioxidant rutin. These findings further highlight the importance of PCCA as a natural antioxidant. In summary, this study shows that purple corn is a plant resource rich in anthocyanidins and potentially has high economic value. Future research will focus on anthocyanidins from purple corn cobs and explore ways to improve their stability in vitro and in vivo.

## 5. Patents

A Chinese national invention patent has been applied for and authorized for the extraction of anthocyanidins from purple corn cobs within this research. The patent number is ZL 202110251959.2.

## Figures and Tables

**Table 1:** Spatial and temporal changes in anthocyanidin content (g/kg, DB) in different parts of purple corn (*Zm* Jizi-01) from Day 70 to Day 120 after sowing. Samples were extracted with mixture of ethanol, water, and hydrochloric acid (2:1:1, *v*/*v*/*v*) and analyzed by HPLC. Different superscript letters indicate significant differences (*p* < 0.05, ANOVA). Statistical comparisons among different parts at the same time were performed using one-way analysis of variance (ANOVA), and different lowercase letters on each value indicate significant differences (*p* < 0.05) among different parts.

Parts	Contents (g/kg, DB)				
	Day 70	Day 80	Day 90	Day 110	Day 120
Stamen	--	--	ND	8.050 ± 0.120 ^b^	--
Leaf	ND ^b^	ND ^c^	0.453 ± 0.015 ^d^	1.918 ± 0.049 ^d^	--
Stalk	0.230 ± 0.010 ^a^	0.211 ± 0.004 ^b^	0.828 ± 0.058 ^cd^	1.565 ± 0.019 ^e^	--
Cornsilk	--	ND ^c^	1.145 ± 0.192 ^c^	3.047 ± 0.227 ^c^	--
Husk	--	7.216 ± 0.675 ^a^	14.204 ± 0.483 ^a^	14.718.4 ± 0.136 ^a^	11.974 ± 0.263 ^a^
Seed	--	--	--	0.389 ± 0.027 ^f^	1.229 ± 0.104 ^b^
Cob	--	--	5.873 ± 0.470 ^b^	11.711 ± 0.375 ^ab^	12.361 ± 0.130 ^a^

‘--’ indicates that the part of the sample was not obtained at the growth stage, and ‘ND’ indicates that there was no anthocyanidin detected in the part.

**Table 2:** Anthocyanidins and anthocyanins of *Zm* Jizi-01 cob after harvest by HPLC and UPLC-QTOF-MS analysis.

No.	Anthocyanidins by UPLC	Anthocyanins by UPLC-QTOF-MS	Major MS/MS by UPLC-QTOF-MS
1	cyanidin	cyanidin-3-glucoside	287, 449
2		cyanidin-3-(dimalonylglucoside)	287, 621
3		cyanidin-3-(6″-malonylglucoside)	287, 535
4		cyanidin-5-(6″-malonylglucoside)	287, 535
5		cyanidin-7-(6″-malonylglucoside)	287, 535
6	pelargonidin	pelargonidin-3-glucoside	271,433
7		pelargonidin-3-(dimalonylglucoside)	271, 605
8		pelargonidin-3-(6″-malonylglucoside)	271, 519
9	Peonidin	peonidin-3-glucoside	301, 463
10		peonidin-3-(dimalonylglucoside)	301, 635
11		peonidin-3-(6″-malonylglucoside)	301, 549

**Table 3:** Scavenging activities of PCCA and control groups against three free radicals.

Sample	IC_50_ (mg/mL)		
	DPPH	ABTS·^+^	·OH
Ascorbic acid	0.015 ± 0.005 ^a^	0.010 ± 0.002 ^a^	0.006 ± 0.003 ^a^
Cyanidin chloride	0.033 ± 0.004 ^b^	0.008 ± 0.007 ^a^	0.009 ± 0.004 ^ab^
Rutin	0.073 ± 0.017 ^c^	0.059 ± 0.024 ^b^	0.016 ± 0.011 ^b^
PCCA	0.159 ± 0.044 ^d^	0.079 ± 0.054 ^c^	0.048 ± 0.034 ^c^

Different lowercase letters represent significant differences among samples as tested by Tukey’s HSD test (*p* < 0.05).

## Data Availability

The original contributions presented in this study are included in the article. Further inquiries can be directed to the corresponding authors.

## Abbreviations

The following abbreviations are used in this manuscript:

PCCA	purple corn cob anthocyanidins
*Zm* Jizi-01	*Zea mays* L., *cv* Jizi-01
DB	dry basis
C3G	cyanidin-3-glucoside
HPLC	high-performance liquid chromatography
UPLC-QTOF-MS	ultra-performance liquid chromatography–quadrupole time-of-flight mass spectrometry
FT-IR	Fourier Transform Infrared Spectroscopy
Pn3G	peonidin-3-glucoside
Pr3G	pelargonidin-3-glucoside
DPPH	1,1-Diphenyl-2-picrylhydrazyl radical
ABTS·^+^	2,2′-Azino-bis(3-ethylbenzothiazoline-6-sulfonic acid) diammonium salt
·OH	hydroxyl radical
